# Cytokines in Spondyloarthritis and Inflammatory Bowel Diseases: From Pathogenesis to Therapeutic Implications

**DOI:** 10.3390/ijms24043957

**Published:** 2023-02-16

**Authors:** Carla Felice, Arianna Dal Buono, Roberto Gabbiadini, Marcello Rattazzi, Alessandro Armuzzi

**Affiliations:** 1 Department of Medicine (DIMED), University of Padova, 35128 Padova, Italy; 2 Unit of General Medicine 1, Ca’ Foncello University Hospital, 31100 Treviso, Italy; 3 IBD Center, IRCCS Humanitas Research Hospital, Rozzano, 20089 Milan, Italy; 4 Department of Biomedical Sciences, Humanitas University, Pieve Emanuele, 20072 Milan, Italy

**Keywords:** cytokine, inflammatory bowel diseases, spondyloarthritis, gut–joint axis, psoriatic arthritis, rheumatoid arthritis

## Abstract

Spondyloarthritis and inflammatory bowel diseases are chronic immune disorders of the joints and the gut that often coexist in the same patient, increasing the burden of each disorder, worsening patients’ quality of life, and influencing therapeutic strategies. Genetic predisposition, environmental triggers, microbiome features, immune cell trafficking, and soluble factors such as cytokines contribute to the pathogenesis of both articular and intestinal inflammation. Most of the molecular targeted biological therapies developed over the last two decades were based on evidence that specific cytokines may be involved in these immune diseases. Despite pro-inflammatory cytokine pathways sharing the pathogenesis of both articular and gut diseases (i.e., tumor necrosis factor and interleukin-23), several other cytokines (i.e., interleukin-17) may be differently involved in the tissue damage process, depending on the specific disease and the organ involved in inflammation, making difficult the identification of a therapeutic plan that is efficacious for both inflammatory manifestations. In this narrative review, we comprehensively summarize the current knowledge on cytokine involvement in spondyloarthritis and inflammatory bowel diseases, underlining similarities and differences among their pathogenetic pathways; finally, we provide an overview of current and potential future treatment strategies to simultaneously target both articular and gut immune disorders.

## 1. Introduction

### 1.1. Epidemiology and Classification

**Figure 1:**
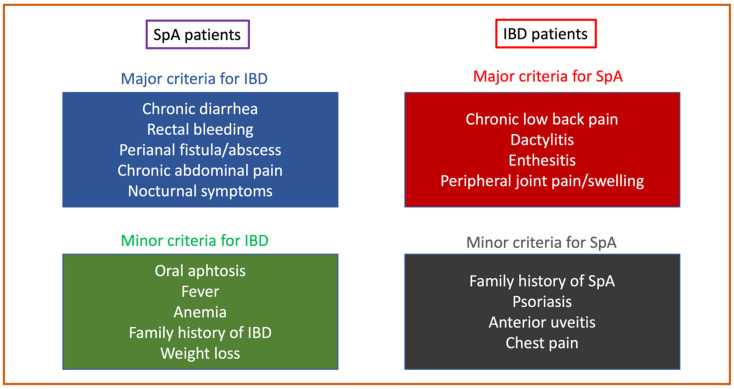
Red flags proposed for referral between gastroenterogists and rheumatologists for early diagnosis of coexisting inflammatory bowel disease (IBD) and spondyloarthritis (SpA) [6].

Spondyloarthritis (SpA) and inflammatory bowel diseases (IBD) are immune-mediated inflammatory disorders (IMID) of the joints and the gut, respectively, which often coexist in the same patient. Their incidence is increasing worldwide, affecting mainly young people, with a significant negative impact on quality of life. The prevalence of SpA varies across geographic areas, ranging from 1.35% in North America (95% CI 0.44–2.79) and 0.54% in Europe (95% CI 0.36–0.78) to lower rates in Asia (0.20%, 95% CI 0.00–0.66), with great heterogeneity due to the mean age of the study populations included in the analyses [1]. The latest reports about IBD prevalence were recently summarized in a perspective from Kaplan and Windsor [2], who identified four epidemiological stages of IBD evolution across countries with different levels of industrialization: emergence, acceleration in incidence, compounding prevalence (where western countries are currently included) and prevalence equilibrium, which represents the future expectation to be achieved in 30–40 years. Currently, the incidence of adult IBD seems to be stable in Western countries, but the overall prevalence will increase with an annual average change of 2.86% (95% CI 2.80–2.92), reaching an estimated prevalence of 981 per 100,000 (95% PI 963–999) in 2030 [3]. The association between IBD and SpA is frequently reported [4], with a prevalence ranging from 18.1 to 47.5% [5]. This makes the management of patients with coexisting articular and gut inflammation more challenging, especially in establishing a therapeutic strategy that is efficacious for both disorders. Collaboration between gastroenterologists and rheumatologists is fundamental in this setting, and there have been important efforts to identify some “red flags” to facilitate appropriate referral and early diagnosis [6] and to create shared algorithms for a combined therapeutic approach (Figure 1) [7,8].

SpA embraces heterogeneous clinical scenarios, which have been defined and classified by the ASAS criteria [9]: in terms of clinical evolution and treatment strategy, it is important to differentiate SpA with involvement of peripheral joints from axial SpA, which is characterized by the inflammation of the spine (ankylosing spondylitis, AS) and/or the sacroiliac joints (sacroiliitis). Psoriatic arthritis (PsA) represents a type of SpA that occurs in patients with psoriasis and mainly involves peripheral joints. All SpA are seronegative, because they do not show the presence of autoantibodies, differently from rheumatoid arthritis (RA), which is a chronic-synovitis-based disorder of peripheral joints that is characterized by the presence of autoantibodies and has an increased risk of association with IBD, as reported in several studies [10,11,12]. Inflammatory bowel diseases also include different pathologies, such as Crohn’s disease (CD) and ulcerative colitis (UC), which are different in terms of gut involvement, evolution, and need for surgery, but may share pathogenetic and therapeutic features.

### 1.2. Overview on Pathogenesis

**Figure 2:**
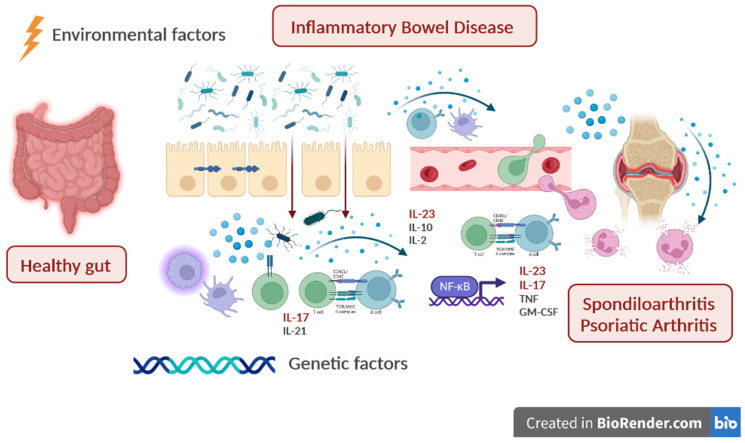
The gut–joint axis in the pathogenesis of IBD and SpA/PsA. Immunological changes in the gut–joint axis: genetic predisposition, environmental factors, and dysbiosis contribute to the loss of intestinal permeability, with consequent activation of immune cells and production of cytokines (i.e., TNF, IL-23, and IL-17), which stimulate chronic inflammation and migration of leukocytes from the gut toward the joints.

Inflammatory bowel diseases and spondyloarthritis have in common several pathogenetic mechanisms, including genetic predisposition [13] and inter-organ communication through cell trafficking (Figure 2) [14]. HLA-B27 has a crucial role in hereditability of ankylosing spondylitis (AS), but such a genetic association is less reported in axial SpA with coexisting IBD [15]. Even patients with psoriasis and PsA have significant genetic susceptibility linked to the MHC (Mayor Histocompatibility Complex) locus (HLA-C(*)06:02), but other genes are also involved, especially those related to cytokine production, the IL-17/IL-23 axis, and the NF-kB and type 2 T helper pathways, which are strongly associated with IBD as well [16]. Immunochip genotype analysis of more than 50,000 patients with seronegative immune-mediated disorders including AS, IBD, and psoriasis confirmed the genetic overlap among these conditions compared to controls [13].

The presence of a gut–joint axis has been demonstrated by several data; in particular, different leucocyte populations that migrated from the intestine to the synovial membranes have been identified [17], and tight junctions were found to be impaired in the mucosa of patients with ankylosing spondylitis (AS) [18,19]. In addition to patients with SpA and a confirmed diagnosis of coexisting IBD, subclinical acute and/or chronic intestinal inflammation was demonstrated in the majority of patients with SpA [20]. The main hypothesis that sustains the gut–joint axis is that the inflammatory process starts in the intestine, where dysbiosis causes increase of permeability and consequent immune activation. In this context, it was recently demonstrated that changes in the intestinal microbiome composition seen in AS and RA may be due to the effect of HLA-B27 and HLA-DRB [21]. The intestinal microbiota, together with genetic, immunological, and environmental factors, have a pivotal role in the pathogenesis of IBD [22], and gut dysbiosis has been demonstrated to be involved as pro-inflammatory trigger even in articular immune diseases. For example, it is now well-recognized that patients with RA [10,23], SpA [24,25], and PsA [26] may have a specific gut microbiota profile. Moreover, in animal models of HLA-B27-positive AS, the disease did not appear in germ-free conditions [27]. Several mechanisms have been described to explain how dysbiosis causes inflammation, including, for example, the interaction with toll-like receptors and antigen-presenting cell activation, induction of T cell differentiation, alteration of intestinal permeability, and the activation of type 3 immunity though the IL-17 pathway [10]. However, despite all these interesting links between gut microbiota and articular inflammation, data on clinical utility of microbiota modulation in this clinical setting are lacking, and a recent placebo-controlled double-blind trial showed no benefit with fecal microbiota transplantation in patients with PsA refractory to methotrexate [28].

Cytokines have a central role in immune disorders and seem to be involved at different levels in the pathogenesis of articular and intestinal IMID, leading to the activation and perpetuation of proinflammatory pathways with consequent tissue damage [29]. Most of the available immune-targeted therapies for the treatment of IBD or SpA have been developed based on specific cytokine involvement in each clinical setting; however, although IMID of different organs may share some inflammatory drivers, there are also several differences, which may challenge the choice of the most appropriate therapeutic strategy, especially when such diseases coexist in the same patient.

### 1.3. Principles of Diagnosis

There is no unique test for the diagnosis of IBD, but gastroenterologists need to integrate clinical, biochemical/fecal, endoscopic, histological, and radiological features, as suggested by international guidelines [30]. Intestinal symptoms in patients with CD may vary, depending on disease location, that may potentially involve every part of the gut from the mouth to the anus, and on lesion severity: chronic diarrhea, recurrent abdominal pain, fever, vomiting, weight loss, and perianal fistula/abscess represent the principal clinical manifestations in CD. The main symptom of patients with UC is chronic diarrhea with rectal bleeding, which can be associated with urgency and abdominal pain. To make a diagnosis of IBD, colonoscopy is mandatory, and multiple biopsies must be taken in all colonic and ileal segments, inflamed or non-inflamed, because microscopic features of chronic inflammation may be present even in apparently normal mucosa [30]. Radiological evaluation of the small bowel, preferably by magnetic resonance (MR) enterography or intestinal ultrasound, offers important information about the activity and the extension of CD, and it is also indicated for evaluating possible complications such as fistulas/abscesses or strictures [31].

The ASAS (Assessment of SpondyloArthritis International Society Group) developed a set of criteria for the diagnosis and classification of peripheral and axial SpA, including a combination of clinical features, such as inflammatory back pain and young age (<45 years), radiological evidence of sacroiliitis, and extraspinal manifestations such as arthritis, enthesitis, uveitis, dactylitis, a concomitant diagnosis of psoriasis or IBD, good response to nonsteroidal anti-inflammatory drugs (NSAIDs), family history for SpA, genetics (HLA-B27), and biochemical markers of inflammation (C-reactive protein) [9]. Based on ASAS criteria, the presence of chronic (>3 months) back pain in subjects of less than 45 years of age can be identified as SpA in the presence of sacroiliitis plus at least one typical SpA feature, or in the presence of HLA-B27 plus at least two other SpA features. Differently from IBD, in which genetic tests are not used in any phase of the disease management (neither for diagnosis nor monitoring or prediction of response to therapies),in the diagnosis of SpA, the assessment of HLA has an important diagnostic role. Peripheral SpA may be identified in patients with arthritis and/or enthesitis and/or dactylitis plus one or more of the other parameters (psoriasis, inflammatory bowel disease, preceding infection, HLA-B27, uveitis, sacroiliitis on imaging) or two or more other parameters (arthritis, enthesitis, dactylitis, inflammatory back pain in the past, family history of SpA) [9].

The diagnosis of associated IBD and SpA is still challenging for rheumatologists and gastroenterologists, who should always collaborate to identify early the clinical features of these diseases. A recent consensus among experts established several “red flags” to facilitate proper referral and to avoid diagnostic delay with possible consequent complications (Figure 1) [6], although they still need to be validated in prospective studies.

The aim of this narrative review was to describe the role and activity of cytokines in SpA and IBD by defining the rationale of immune-targeted biological therapies in each condition and underlining similar and different cytokine pathways involved. The specific roles of cytokines in the pathogenesis of IBD and SpA are also described, and the possible evolution of pro-inflammatory pathways during disease progression and biological therapies are summarized. We have also dedicated a section on the most recent evidence on dual targeted therapy, which is based on the combination of two biologics with different molecular targets and represents the newest therapeutic approach in patients with particularly aggressive phenotypes and/or with coexisting immune disorders.

## 2. Cytokines in Spondyloarthritis

Tumor necrosis factor alpha (TNFα) was the first cytokine targeted by a biological agent in patients with inflammatory arthritis. It is secreted by several immune cell types, such as neutrophils, T cells, and macrophages, recruiting further inflammatory cells and stimulating the production of other cytokines. The central role of TNFα in the pathogenesis of immune arthritis was historically elucidated decades ago, when it was demonstrated that TNFα and its receptors are abundant in the synovial membrane of patients with RA [32]. TNFα produced by cultured mononuclear cells from the joints of patients with RA is also a key mediator to stimulate other pro-inflammatory cytokines [33]. Many cell types in the joint, including macrophages, fibroblasts, T lymphocytes, and the vascular endothelium, could also produce and react to TNFα in both an autocrine and paracrine way [34]. Such findings formed the basis for the clinical use of monoclonal antibodies directed against TNFα for the treatment of RA and other forms of immune arthritis, starting from infliximab, the first chimeric anti-TNFα, and further arriving at similar biological agents, such as adalimumab, golimumab, certolizumab, and etanercept. All these anti-TNFα are currently approved for the treatment of RA, axial spondyloarthritis, and psoriatic arthritis, with similar long-term efficacy rates and safety profile. TNFα interacts also with non-inflammatory cells, such as joint fibroblasts, contributing to chronic tissue damage and fibrosis: at this regard, the early use of anti-TNFα demonstrated to be able to prevent radiological progression and to protect joint integrity in patients with RA [35]. Moreover, TNFα is an important activator of osteoclasts, leading to bone erosions in chronic arthritis [36] and contributing to osteoporosis; therefore, its blockage in this clinical setting may ameliorate bone metabolism and further reduce tissue damage [37].

In the pathogenesis of SpA, and in particular AS, the main key player is the activation of ciclooxygenase-2 after mechanical stress response [38], which increases the prostaglandin-E levels and then induces the overproduction of interleukin (IL)-17A, the principal cytokine involved in AS. IL-17A is produced by several immune cell types, including CD4 + Th17 lymphocytes, CD8+ cytotoxic T17 cells, T γ/δ lymphocytes, and innate lymphoid cells type 3 (ILC3), which are all present in the tendons and in the entheses of the spine cord and whose interactions determine chronic inflammation [39]. In addition to TNFα, IL-17A also stimulates osteoblast activity, leading to an exacerbated bone response to pro-inflammatory stimuli and causing subsequent tissue damage [40]. Moreover, IL-17 is a pain mediator that significantly contributes to symptoms in patients with AS [41]. The use of monoclonal antibodies directed against IL-17A, such as secukinumab or ixekizumab, have been demonstrated to be effective in the treatment of AS, and their use is currently widely approved [42,43].

Psoriatic arthritis (PsA) is a further immune articular disease that is usually associated with psoriasis, but is also common in other inflammatory conditions, such as IBD. It mainly affects peripheral joints, differently from AS, although an axial involvement may occasionally be present. Patients with PsA may be successfully treated with IL-17A inhibitors, as well as those with AS, confirming an important pathogenetic role of IL-17A in this setting [44,45]. However, in PsA, the IL-17A pro-inflammatory pathway seems to be dependent on IL-23, which comes from inflamed skin or gut and activates the cells that secrete IL-17 (T17, ILC3, and T γ/δ lymphocytes) leading to articular damage [29], whereas in AS, the production of IL-17A is stimulated directly by COX-2 and prostaglandin E, independently of IL-23. Such differences may justify the fact that IL-23 antagonists (i.e., ustekinumab, a monoclonal antibody against the subunit p40 of IL-12 and IL-23) show low rates of success in patients with predominantly axial involvement and are efficacious in those with peripheral PsA [46,47].

Other cytokines seem to have specific pathogenetic roles in articular diseases. For example, IL-6 represents a crucial cytokine in patients with RA, having regulatory effects on the intra-articular leucocyte infiltrate and potentially influencing B lymphocytes and auto-antibody production [29]. IL-6 is overexpressed in both synovial fluid and membranes of patients with RA [48], and interacting with its receptor can activate neutrophils, T cells, B cells, monocytes, and osteoclasts. Tocilizumab and sarilumab, two monoclonal antibodies directed against the receptor of IL-6, showed to be efficacious for the treatment of patients with RA [49,50], whereas they were not effective in AS [51] and were not tested in PsA. Similarly, IL-1 is a different cytokine that is involved in the pathogenesis of RA and the inhibition of its pathway using a monoclonal antibody (canakinumab) [52] or anakinra (a human recombinant IL-1Rα antagonist) [53] has been demonstrated to be effective in this clinical setting. IL-1 inhibition showed significant efficacy in other rheumatological disorders as well, such as systemic juvenile idiopathic arthritis and adult-onset Still’s disease; however, no data are available in patients with PsA or AS so far.

Apart from the direct cytokine inhibition through specific biological agents, a valid therapeutic strategy in several IMID is the blockage of intracellular signal transduction pathways downstream of cytokines, which represents the mechanism of action of Janus kinase (JAK) inhibitors, such as tofacitinib, baricitinib, filgotinib, and upadacitinib, which have been already approved for the treatment of rheumatological conditions. In particular, JAK inhibitors target the intracellular signal transduction shared by many pro-inflammatory pathways, with a final simultaneous inhibition of several cytokines. At the same time, depending on the principal JAK inhibited (JAK1, JAK2, JAK3, or tyrosine kinase (TYK) 2), each drug may primarily impact specific cytokines; for example, IL-6 is mainly targeted by JAK1, JAK2, and TYK2 inhibition, but not JAK3; also, IL-23 is mainly affected by JAK2 and TYK2 inhibition [54]. In patients with immune arthritis, the reduction of joint pain represents an important clinical outcome: interestingly, a modulation of nociception has been demonstrated with the use of JAK inhibitors in patients with RA and PsA, in particular with tofacitinib and baricitinib, and this effect also seems to be due to cytokine modulation [55].

## 3. Cytokines in Inflammatory Bowel Diseases

Several cytokines are crucial in the activation and perpetuation of pro-inflammatory pathways in the intestinal mucosa of IBD patients. TNFα was one of the first cytokines that was shown to have a main role in the induction of intestinal inflammation. Animal models of colitis that were genetically modified to have an enhanced production of TNFα (TNF^ΔARE^ mutant mice) developed transmural chronic inflammation with non-caseating granulomas, mimicking macroscopic and histological alterations observed in patients with CD [56]. TNFα is a pleiotropic cytokine that interacts with several intestinal cell types (endothelial, epithelial, and immune cells) and induces inflammation through multiple pathways, modulating, for example, intestinal epithelial cell growth and apoptosis, gut permeability, and mucosal integrity via matrix metalloproteinase (MMP) production [57]. Together with IL-2 and interferon (IFN) γ, TNFα also drives pathogenic T helper (Th)1-like responses that characterize the immune profile of active CD [58]. The publication of almost all findings on the pathogenetic role of TNFα occurred in the nineties, during the same period of the first demonstrations of infliximab efficacy in the treatment of IBD patients. Since then, adalimumab, certolizumab (only in the United States) and golimumab (only for UC) have also been approved for the treatment of IBD. Interestingly, the use of etanercept, a dimeric fusion protein that also targets TNFα and is currently approved for the treatment of several articular immune disorders, is not effective in CD patients [59] and seems to have negative effects on the intestine, inducing or worsening gut inflammation [60]. Etanercept, differently from other biologics, targets only soluble TNFα and not that bound on the membrane of immune cells, such as macrophages, which was demonstrated to stimulate both T-cell cytokine production and T-cell survival in patients with IBD [61]. Therefore, the blockage of the same cytokine, such as TNFα, may potentially induce different effects in each IMID, depending on which specific pathway is affected, with consequent important therapeutic implications.

The active involvement of IL-23 in IBD pathogenesis was first demonstrated at a genetic level. In 2006, a genome-wide study found a strong association among coding and non-coding IL-23R variants and both CD and UC, suggesting that such signaling pathways could be further therapeutic targets in IBD [62]. Subsequent experiments on an animal model of colitis confirmed that neutralization of IL-23 significantly ameliorates colonic inflammation [63]. IL-23 is mainly produced by macrophages and dendritic cells in the intestinal mucosa and may alter the balance between pro- and anti-inflammatory pathways, stimulating Th1 and Th17 cells and inhibiting regulatory T cells [64]. The use of ustekinumab, which targets the subunit p40 of both IL-23 and IL-12, is efficacious in the treatment of IBD, although the role of IL-12, which is normally involved in Th1 differentiation, seems to be marginal in this setting. The predominant function of IL-23 compared to IL-12 in IBD was confirmed by recent data about the use of new selective IL-23 antagonists. Risankizumab, for example, a monoclonal antibody directed against the subunit p19 of IL-23, proved to be efficacious in inducing and maintaining clinical remission in CD patients [65,66]. Successful results were obtained even with guselkumab, another selective p19 IL-23 antagonist, as induction therapy for patients with CD [67]. IL-23 is strictly related to the IL-17 family, especially in immune arthritis, as previously described in this review. However, the IL-17A seems to have a protective role in the gut, principally due to its ability to promote epithelial integrity and antimicrobial defense in the intestinal mucosa. Maxwell et al. demonstrated that the selective inhibition of IL-17A leads to colitis exacerbation in animals, mainly due to altered epithelial permeability and function; in the same model of colitis, treatment with inhibitors of p40 or p19 subunits showed a significant and comparable reduction of the intestinal inflammation [68]. Moreover, the mucosal production of IL-17A is provided by γ/δ T cells but independently from IL-23, leading to a normal level of intestinal IL-17A even during therapy with antagonists of IL-23 [69]. In line with these data, the treatment of CD patients with IL-17 antagonists, such as secukinumab, showed negative results in terms of both efficacy and safety [70], and several cases of new onset UC were reported during therapy with IL-17 inhibitors for rheumatological indications [71]. The axis IL-17/23 represents a further example of different organ-selective signaling pathways of the same cytokine, based on specific environmental interactions.

Other cytokines, such as IL-6, currently have a minor role in the clinical management of IBD, despite their active involvement in the pathogenesis of the diseases. The level of IL-6 is significantly increased in the intestinal mucosa of IBD patients, and its blockage by a specific monoclonal antibody led to good rates of clinical response in refractory CD but increased the incidence of serious adverse events, probably due to a strong immunosuppression [72]. Recently, a new IL-6 inhibitor has been described by Schreiber et al.: olamkicept is assembled by two complete extracellular domains of gp130 (the IL-6/IL-6R signal transducer), which are dimerized by fusion to the fragment crystallizable (Fc) region of a human IgG1 and act on IL-6 trans-signaling, therefore specifically blocking chronic inflammation without interfering with normal defense activities involving overall IL6 signaling [73]. The results of a 12-week open-label phase 2 trial demonstrated good rates of clinical response and remission in a patient with IBD treated with olamkicept, which was also well-tolerated [73]; however, larger controlled studies are needed to confirm these preliminary findings.

Differently from CD, UC is characterized by the activation of Th2 pathway in the colonic mucosa, also recruiting Th9 cells, ILC2, and eosinophils under the stimulus of IL-33 and with subsequent production of other specific cytokines, such as IL-5, IL-6, IL-9, and IL-13 [74]. However, a therapeutic attempt with selective IL-13 inhibition did not show any benefit in patients with UC [75,76].

## 4. Cytokine-Based Therapeutic Scenarios in IBD and SpA

All therapeutic scenarios for patients with IBD and SpA, when analyzed as distinct diseases, may benefit from the results of several controlled clinical trials in which cytokine-targeted therapies were specifically studied in each intestinal or articular clinical setting. Unfortunately, controlled trials including patients with associated IBD and SpA that focus on combined primary outcomes have yet to be conducted. Therefore, to manage patients with coexisting chronic inflammatory intestinal and rheumatological diseases, some therapeutic strategies have been proposed, principally by the consensus of experts, based on shared pathogenetic pathways and considering drugs with documented efficacy on both clinical entities [7,8,77]. Anti-TNFα and IL-23 inhibitors currently represent the main cytokine-based therapeutic strategies for patients with coexisting IBD and SpA, except for etanercept, which is not indicated in IBD, and preferring the use of ustekinumab in peripheral but not axial SpA. In contrast with selective cytokine targets, JAK inhibitors are efficacious for the treatment of several IMID, including immune arthritis and IBD, with a mechanism of action based on the simultaneous block of different cytokine pathways [78]; therefore, they may represent a further valid therapeutic option in patients with associated IBD and SpA.

The first evidence of treating IBD and SpA with the same cytokine-targeted therapy was with anti-TNFα, although randomized controlled trials with a pre-specified combined outcome were never realized, and there are very few interventional studies confirming the efficacy of both infliximab and adalimumab for treating articular disorders in IBD [79]. The majority of data come from retrospective analyses: Vavricka et al., for example, described the outcomes of a large Swiss cohort of IBD patients, showing high percentages of response for extraintestinal symptoms (71.8%) during treatment with infliximab [80]. The overall efficacy of anti-TNFα in treating simultaneously different diseases was confirmed in a comprehensive systematic review [79], which also included in the analysis several open-label studies on articular extraintestinal manifestations in IBD. Recently, retrospective real-life experiences have been reported analyzing clinical outcomes in IBD patients treated with other biological therapies for an extraintestinal indication: in a retrospective multicenter Italian study, Pugliese et al. described a cumulative probability of maintaining ustekinumab treatment (defined by sustained IBD clinical benefit) of 97.1% at 6 months and 77.1% at 12 months in IBD patients who started this therapy for dermatological or rheumatological indications (psoriasis or PsA) [81]. Liefferinckx et al. described a retrospective Belgian cohort of 152 CD patients who received ustekinumab as second- or third-line biological therapy; at baseline, approximately 30% presented arthralgia and 11.2% had a concomitant diagnosis of AS, and among them, 54.4% and 82.6% had a complete resolution of articular symptoms at week 16 and week 52, respectively. However, patients with a diagnosis of AS had no benefit for arthralgia during therapy with ustekinumab, confirming previous data from clinical trials [82]. Further retrospective findings in CD patients treated by ustekinumab were reported by Biemans et al., who described more than 50% articular remission at a median follow-up of 52 weeks [83]. Positive results of ustekinumab for the specific treatment of extraintestinal manifestations were also reported in small cohorts of IBD patients [84]. Interestingly, a post-hoc analysis of the UNITI trials showed no differences in arthritis/arthralgia resolution at week 52 in patients treated with ustekinumab (69%) compared to placebo (61.1%, *p* = 0.258) [85]. In an international (Belgian and Spanish) multicenter retrospective study, all IBD patients treated with ustekinumab or vedolizumab during the previous 10 years were included, and new onset or deterioration of pre-existing arthropathy (defined by the presence of joint inflammation) was analyzed over a 2-year follow-up, showing comparable results between the two drugs [86]. Studies on extra-articular outcomes in patients with rheumatological conditions treated with biologics are still lacking. Retrospective data showed that a concomitant diagnosis of IBD does not affect treatment persistence when patients with AS are treated by anti-TNFα [87]. In another large population of patients with axial SpA (n = 2420) recruited from 83 centers across the United Kingdom, a concomitant diagnosis of IBD led to more frequent prescription of adalimumab and less frequent prescription of etanercept compared to other biologics [88]. However, robust data about the efficacy of specific cytokine-targeted therapies for the treatment of both articular and intestinal immune disorders are not available yet.

In clinical practice, there are no reliable predictors of response to specific drugs; therefore, treatment strategies are usually chosen depending on patients’ clinical history, activity of the disease, safety profile, and costs. During maintenance therapy with biologics, some patients may lose benefit or develop paradoxical reactions, including cutaneous and articular inflammatory disorders, probably because the inflammatory cytokine pathways involved at the beginning may change over time. Interestingly, there is an emerging hypothesis that secondary loss of response or refractoriness to a biological treatment may be due to an “escape” mechanism in the targeted cytokine: for example, Schmitt et al. analyzed mucosal and blood cells of CD patients before and during anti-TNFα treatment, showing up-regulation of mucosal IL-23p19, IL-23R, and IL-17A in non-responders compared to responders and identifying intestinal IL-23R+ T cells resistant to apoptosis which may explain the refractoriness to anti-TNFα [89]. These findings confirm that IL-23 may induce an escape mechanism in CD patients receiving anti-TNFα, and therefore, the use of ustekinumab may represent the best option in this setting. Alivernini et al. analyzed the histological characteristics of inflammatory infiltrate in the synovia and in the gut of IBD patients who developed paradoxical arthritis during anti-TNFα therapy, showing similar immune cells (CD68+, CD3+, CD117+, and CD20+) in both tissues; they also confirmed that swapping to an anti-IL23 may be more effective than switching to a different anti-TNFα to treat such extraintestinal disorders [90]. The development of paradoxical reactions during biological treatment is less studied from a pathogenetic point of view. However, a diagnosis of SpA or psoriasis during, for example, anti-TNFα maintenance therapy for IBD implies an empiric change of the therapeutic management, adding an immunomodulator or swapping to a different biological class. Currently, there are no blood or tissue biomarkers that may help to identify the treatment option with the best probability of success in this clinical context.

## 5. Dual Targeted Therapy

The combination of biological agents with different molecular targets (dual targeted therapy, DTT) is an emerging therapeutic strategy used to reinforce the therapy of one immune disease or to treat two different pathologies, such as IBD with associated SpA. There are several hypotheses that sustain the rationale to use a DTT: first, the combination of two treatments with different targets may increase the rates of success; also, it may reduce the risk of “escape mechanisms” that may potentially lead to loss of response; moreover, it may offer the possibility to treat at the same time two different diseases with a specific pathogenetic background. A few trials have investigated the efficacy of DTT to treat the same disease, especially in rheumatological disorders, but with conflicting results. For example, association of anakinra and etanercept did not add any benefit compared to etanercept alone in patients with RA refractory to methotrexate [91]. More recently, the use of ABT-122, which is a novel dual variable-domain immunoglobulin against both TNFα and IL-17A, led to clinical outcomes similar to adalimumab in monotherapy in patients with RA [92]. Apart from efficacy data, a further concern for the use of DDT is safety, based on the assumption that simultaneous inhibition of several cytokine pathways may worsen the immunosuppression and therefore increase the risk of immune-related side effects. In a recent review and metanalysis by Boleto et al. [93], side effects reported in five trials on biological combination therapy in patients with RA (410 treated with double biologics compared to 213 on monotherapy) were significantly more frequent in those who were treated by combination therapy compared to monotherapy (94.6 vs 89.1%, OR 2.07, 95% CI 1.11–3.86), and serious infections in particular (6.7 vs 0.6%, OR 5.58, 95% CI 1.25–24.90). The main controlled trials on DTT in patients with RA are described in Table 1.

Evidence on DTT efficacy in IBD patients is also increasing, but is mainly based on small cohort studies or case series. A recent systematic review and meta-analysis found that the prevalent indication for DTT (two biologics or one biologic + JAK inhibitor) in IBD patients is refractory disease (81%), whereas extraintestinal manifestations account for 12% of all indications [94]; among all possible combinations, anti-integrin (vedolizumab or natalizumab) was the biologic most frequently associated with other drugs (anti-TNFα, anti-IL12/23, or JAK inhibitors), probably due to its gut selectivity and safety profile. For IBD disease activity, the rates of clinical and endoscopic remission were 52% and 33%, respectively, whereas in patients with baseline active extraintestinal manifestations, the response rate was 49.9% (95% CI, 14.2–85.7%) [94]. Safety outcomes reported rates of 35% and 6% for any adverse events or serious adverse events, respectively [94]. Similar efficacy and safety results were described in a further meta-analysis by Alayo and co-authors [95]. In pediatrics as well, the first encouraging data are beginning to emerge: Dolinger et al. described positive outcomes with 75% steroid-free remission at 6 months in 16 young IBD patients treated with several combinations of DTT (not including an anti-TNFα) [96]. After a first controlled randomized trial published in 2007 that included CD patients who were treated by a combination of anti-TNFα and natalizumab (an anti-integrin approved for CD only in the United States) [97], there were no additional controlled trials on DTT in IBD until 2022 (Table 1): in adult patients with UC, a combination of guselkumab (anti-IL-23) and golimumab (anti-TNFα) for induction of remission was compared to golimumab or guselkumab alone, followed by maintenance with guselkumab in monotherapy. Differently from the rheumatological experience, the preliminary results of this trial in UC patients showed higher benefit from the combination regimen in achieving the primary outcome (clinical response at week 12) compared to golimumab monotherapy, whereas no difference was found between combination and guselkumab alone; in terms of clinical remission and endoscopic response at week 12 (secondary outcomes), the dual treatment was more effective than both monotherapy regimens [98]. Two studies on 48-week outcomes in CD and UC patients (DUET trials) are currently ongoing that are comparing the combination of guselkumab and golimumab to each monotherapy (ClinicalTrials.gov Identifier: NCT05242471 and NCT05242484).

Prospective controlled studies on DTT for the treatment of IBD and concomitant extraintestinal manifestations are lacking, and the available data are currently based on case series [99]; therefore, it is difficult to find the proper balance between the benefits, risks, and costs of such combined therapeutic approaches. In term of safety, vedolizumab, an anti-integrin that selectively inhibits the migration of leukocyte to the gut, is a further biologic agent approved for the treatment of IBD with potential minimum effects on other organs. Therefore, to potentially reduce the immunosuppression-related side effects of DTT, vedolizumab may represent a valid option in association with another biologic in patients with SpA associated with IBD who do not respond to other therapies. Hypothetically, the concomitant use of IL-23 inhibitors and anti-TNFα may also reduce the possibility of paradoxical immune reactions, which are not so rare. The absence of controlled trials in these settings represents an important limitation; however, the EXTRA consensus, organized by the International Organization for the Study of Inflammatory Bowel Diseases, made an important effort to identify simple methods to measure clinical outcomes of extraintestinal manifestation in trials including primarily IBD patients [100]: such guidelines may represent a valid basis for the future development of controlled trials specific to these clinical scenarios.

**Table 1:** Randomized controlled trials on dual targeted therapy in inflammatory bowel diseases and rheumatological immune disorders.

Reference	Year	Disease	N Patients	Combination	Control Arm	Outcome	Safety	Follow-up (Months)
Sands et al. [97]	2007	CD	79	Natalizumab + infliximab	Infliximab monotherapy	Safety	No differences in AEs	10 weeks
Sands et al. [98]	2022	UC	214	Guselkumab + golimumab	Guselkumab or golimumab monotherapy	Clinical response (Mayo score) at week 12	No differences in AEs	3
Genovese et al. [91]	2004	RA	242	Etanercept + anakinra	Etanercept monotherapy	ACR 50 at week 24	Increased rate of AEs in combo therapy	6
Weinblatt et al. [101,102]	2006	RA	167	Abatacept + TNFi abatacept + anakinra	TNFi or anakinra monotherapy	Safety	Increased rate of AEs in combo therapy	12
Weinblatt et al. [102]	2007	RA	121	Abatacept + etanercept	Etanercept monotherapy	ACR 20 at 6 months	Increased rate of AEs in combo therapy	12
Blank et al. [103]	2009	RA	18	Rituximab + etanercept	Rituximab monotherapy	Safety	No differences in AEs	8
Greenwald et al. [104]	2011	RA	51	TNFi + rituximab	TNFi monotherapy	Safety	No differences in AEs	6
Glatt et al. [105]	2019	RA	79	Certolizumab + bimekizumab (anti-IL-17A and IL-17F)	Certolizumab monotherapy	DAS28 (CRP) and safety	Increased rate of AEs in combo therapy	4
Genovese et al. [92]	2018	RA	222	ABT-122 (dual TNF and IL-17A inhibitor)	Adalimumab	ACR20 at week 12	No differences in AEs	3
NCT00845832 [106]	2013	RA	24	Rituximab + tocilizumab	Tocilizumab monotherapy	LDA at week 16	No differences in AEs	12

CD, Crohn’s disease; UC, ulcerative colitis; RA, rheumatoid arthritis; ACR, American College of Rheumatology; TNFi, tumor necrosis factor inhibitor; DAS, Disease Activity Score; CRP, c-reactive protein; IL, interleukin; AE, adverse event; LDA, low disease activity.

## 6. Conclusions

The pathogenetic role of cytokines in IBD and SpA is well-defined, but new pathways and thus new possible therapeutic targets are still emerging, making the scenario even more complex. The knowledge about the exact mechanisms involved in chronic tissue damage and about the molecular targets of available therapies is fundamental to establishing the correct treatment algorithm for each clinical setting. It is crucial to recognize that cytokine interplay may be disease-specific, tissue-specific, and may also change over time in the same patient. The identification of predictors of response to specific therapies and in each single patient should be the main objective of future therapeutic interventions to identify the best personalized approach. The lack of controlled data about the cotreatment of rheumatological and intestinal immune disorders remains an important unmet gap of knowledge.

## Data Availability

Data sharing not applicable. No new data were created or analyzed in this study. Data sharing is not applicable to this article.
